# Anti-plasmodial activities of *Combretum molle* (Combretaceae) [Zwoo] seed extract in Swiss albino mice

**DOI:** 10.1186/s13104-018-3424-4

**Published:** 2018-05-18

**Authors:** Merkin Anato, Tsige Ketema

**Affiliations:** Department of Biology, College of Natural Sciences, Jimma University, Jimma, Ethiopia

**Keywords:** Acute toxicity, Anti-plasmodial, *Combretum molle*, Medicinal plant

## Abstract

**Objective:**

Objective of the study was to evaluate in vivo anti-plasmodial activities of *Combretum molle* seed extract.

**Methods:**

As a standard protocol, initially the acute toxicity of the plant seed extract was checked following single administration of crude seed extract of the plant at doses 500, 1000 and 2000 mg/kg. This was followed by evaluation of anti-plasmodial activity of crude seed extract of the plant following a 4 days suppressive test.

**Results:**

In acute toxicity study sign of toxicity was not observed. Also physical and behavioural changes were not detected. The crude seed extract of *C. molle* showed, 63.5% parasite suppression in mice infected with *Plasmodium berghei* ANKA (*Pb*A) murine parasite and treated with 250 mg/kg of seed extract of *C. molle*. Relative survival time of mice treated with 250 mg/kg showed significantly longer survival than the negative control, while lower than mice treated with the standard drug, chloroquine. The plant seed extract on day-4 post-infection showed significant (P < 0.05) protection against body weight reduction, high body temperature and hemolysis of RBC at relatively lower doses. At optimum dose the crude extract of *C. molle* seed has good chemo-suppressive activity against *Pb*A parasite and improved some clinical symptoms of malaria in mice.

## Introduction

Although the current prevalence and incidence of malaria showing a declining pattern globally, still it is a major public health problem for nations in developing regions. The major causes of the concern is, the frequent antimalarial drug resistance trait evolving in the plasmodium parasite, which is compromising the effective treatment of the disease. Thus, there is a pressing need for anti-malarial drug discovery research that will provide a new substance with unique mode of action, effective, safe, and affordable for the needy people [1]. With this regard, plants are a potential source of new antimalarial drugs, since they contain a number of metabolites with a great variety of structures and pharmacological activities [2].

According to the earlier studies on antimalarial activity, several traditionally claimed Ethiopian medicinal plants showed promising antimalarial activity [3]. This is because most of these plants are rich in secondary metabolites [4]. *Combretum molle* is a popular medicinal plant widely used in Africa for treatment of different aliments including malaria and HIV [5–7]. Similarly, seed of this plant is widely used for treatment of malaria by residents in rural areas of Ethiopia, mainly in Gambella regional state. Although seed of *C. molle* plant is widely used for treatment of malaria, to the best of our knowledge, its in vivo anti-plasmodial activity is not evaluated yet. Therefore, aim of this study was to assess the anti-malarial activity of seed of the *C. molle* plant using mice as animal model.

## Main text

### Methods

The seed of *C. molle* was collected from Gambella Region, Agnua zone (about 886 km south west of Addis Ababa), Ethiopia. The plant seed was garbled, dried in the processing room, and powdered using grinding machine, and then kept at room temperature in a well-closed and amber coloured bottle until extracted. The dried and powdered plant material was extracted by maceration using methanol (Sigma Aldrich). Then methanol solvent was allowed to evaporate. Swiss albino mice, both sex, age 8–10 weeks, and weighed of 31–42 g were obtained from Ethiopian Public Health Institute (EPHI), Addis Ababa Ethiopia. They were kept at temperature of 22 ± 3 °C, relative humidity of 40–50% and 12 h light/12 h dark cycle. The mice were allowed to adapt to the laboratory environment for a week before being subjected to the experiments.

Acute toxicity of the extract was checked using three doses, 500, 1000 and 200 mg/kg extract following the standard procedure applied by Merkin et al. [8]. Test animals were observed for 2 weeks for any signs of toxicity such as loss of appetite, hair erection, convulsions, salivation, diarrhea, and mortality.

For in vivo anti-plasmodial activity tests a 4-day suppressive methods was used. For this particular assay, mice were blindly randomized into four groups in duplicate: the first two groups were treated with 125 and 250 mg/kg seed extract; the second two groups were (i) untreated *Plasmodium berghei* ANKA (*Pb*A) infected control (negative control) and (ii) *PbA* infected chloroquine (positive control) treated respectively. Accordingly, CQ sensitive strain of *P. berghei* ANKA (*Pb*A) parasite was used to infect mice for the 4-day suppressive test. Donor mice carrying 20–30% parasitemia of *Pb*A was obtained from EPHI. Infected blood sample collected from the donor mice were diluted in physiological saline solution to 5 × 10^7^ infected RBCs [9]. Mice were intraperitoneally inoculated with ~ 0.2 mL of the diluted infected blood on the 1st day (D0). Three hours after infection with *Pb*A, mice were orally administered with 125 and 250 mg/kg/day doses of the *C. molle* seed crude extract. CQ (Adigrat Pharmaceutical Factory, Ethiopia) at the dose of 10 mg base/kg/day and an equivalent volume of vehicle [0.5 mL 20% of Tween 80 in saline (Sigma Aldrich)] was administered to the positive and negative control groups respectively, for 4 consecutive days. Some clinical symptoms and physicals changes of each mouse in all the groups were monitored.

On the 5th day (D4), thin smears were made from the tail nip blood (collected by applying local anesthesia on the tail). The smear fixed with methanol and stained with 10% Geimsa pH 7.2 for 15 min. The duplicate stained slides was examined under a microscope (100× magnifications). The parasitemia level was determined by counting the number of parasitized RBCs out of 100 in random fields of the microscope [10]. Mean percentage of parasite suppression was calculated following a standard protocol [11].

The capillary tubes were filled with blood up to 3/4th of their volume and seals at the dry end with sealing clay to determine pack cell volume (PCV). The tubes were then placed in a micro-haematocrit centrifuge with the sealed end outwards and centrifuged for 5 min at 11,000 rpm. The tubes were then taken out of the centrifuge and PCV was determined.

Mean survival time for each group was determined arithmetically by calculating the average survival time (days) over a period of 30 days. Mice were monitored daily and the number of days from the time of inoculation of the parasite up to death was recorded for each mouse in the treatment and control groups throughout the follow up period. Then mean survival time for each group was calculated.

In survival study, mice were systematically euthanized when they were found in a waning state in accordance to Restagno et al. [12]. Classical signs of distress such as anorexia, weight loss, bending, prostration, breathing difficulty, ruffled haircoat, dehydration, were assessed. Mice showing at least four of these criteria were humanely euthanized via ketamine anesthesia followed by cervical dislocation. Mice exhibiting less than four of these criteria were further waited again for 8 h and if the symptoms worsened they would be euthanized. Those mice survived the study and the controls, were also euthanized after the study was completed. Euthanized mice were considered as dead.

### Data analysis

Data was analysed using SPSS software, version 20.0 and the results were expressed as mean ± standard deviation (SD). One-way analysis of variance (ANOVA) was used to compare results and followed by Turkey’s HSD post hoc test. Statistical significant level was considered at P < 0.05.

### Results

Mean percentage of infected RBCs of mice infected with *Pb*A and subsequently treated with seed crude extract of *C. molle* (125 and 250 mg/kg) showed significant (P < 0.05) reduction of parasitemia. The methanol extract of seed of the *C. molle* at 250 mg/kg exhibited an inhibition of 63.5%, on day 4-post infection (p.i.). The percentage inhibitions of the parasite was significantly (P < 0.05) higher for mice treated with 250 mg/kg than the negative control and those mice received lowest dose (125 mg/kg) of the extract, while significantly (P < 0.05) lower than those treated with the standard drug (CQ). As the dose of the seed extract increased, its parasite suppression potential was increased. Although, the survival time of the seed extract treated group (250 mg/kg) p.i. was significantly (P < 0.05) higher (12 days) than the negative control (9 days), but it was much shorter than that of the standard drug treated group (Table 1).

**Table 1:** Percent parasitemia, percent parasite suppression and mean survival time of mice infected with *Pb*A and treated with seed crude extracts of *C. molle*

Groups	Anti-malarial activities of the extract on day 4 p.i.		Survival time (mean day ± SD)
	% parasitemia (mean ± SD)	% parasite suppression/inhibition	
Control	35.7 ± 1.51	0.0	9.5 ± 0.29
125	26.9 ± 1.88^a1^	36.6 ± 2.72^a2^	10.5 ± 0.11
250	15.5 ± 1.9^a2^	63.5 ± 3.49^a1^	12^a^ ± 0.56
CQ	6.28 ± 0.27^b3^	85.23 ± 0.04^b2^	> 30

NB: ^a^ compared to control, ^b^ compared to chloroquine (CQ) treated (10 mg/kg); ^1^ P < 0.05; ^2^ P < 0.01, ^3^ P < 0.001

*CON* control, *p.i.* post-infection

The comparative analysis on packed cell volume (PCV) of mice treated with the seed extracts (under all doses) was significantly (P < 0.05) reduced on day 4 p.i. than mice group treated with CQ on the same day, but significantly increased (P < 0.05) than the negative control. Similarly, protection of the seed extract from body weight reduction showed significant improvement in mice groups treated with different doses of seed extract of *C. molle* than negative control. But did not showed significant differences from CQ treated mice. On 4-day p.i. of *Pb*A infection increment of rectal temperature was recorded in all mice treated with different doses of the extract, while it was reduced in CQ treated mice group. However, mice groups treated with 125 and 250 mg/kg seed extract of the plant showed significantly (P < 0.05) lower rectal temperature than the negative control (Table 2).

**Table 2:** Temperature, weight and packed cell volume of infected mice treated with crude extract of seed of *C. molle* in the 4 day suppressive test

Animal Group	Packed cell volume			Body weight			Temperature		
	PCV-D0	PCV-D4	% change	BW0	BW4	% change	T0	T4	% change
Control	63.2 ± 0.97	49.6 ± 0.19	− 21.52	33.8 ± 0.63	28.6 ± 0.2	− 15.38	35.84 ± 0.54	36.7 ± 0.77	2.4
125	61.2 ± 0.78	51.6 ± 0.67	− 15.7^b2^	36.4 ± 0.47	33.6 ± 0.4^a1^	− 7.69	36.88 ± 0.19	37.02 ± 0.13	0.38^a2^
250	62.8 ± 0.09	51.2 ± 0.68	− 18.47^b2^	33.8 ± 0.96	32.9 ± 0.33^a2^	− 2.66	36.54 ± 0.16	36.62 ± 0.52	0.22^a2^
CQ	57 ± 0.68	53.5 ± 0.65	− 6.14	34.5 ± 0.41	32 ± 0.16	− 7.24	37.6 ± 0.34	36.45 ± 0.45	− 3.05

Data are expressed as mean ± SD; ^a^ compared to control, ^b^ to chloroquine (CQ) 10 mg/kg; ^1^ P < 0.05; ^2^ P < 0.01

*D0* pre-treatment value on day 0, *D4* post-treatment value on day 4, −ve sign shows reduction, +ve sign shows an increment

**Fig. 1:**
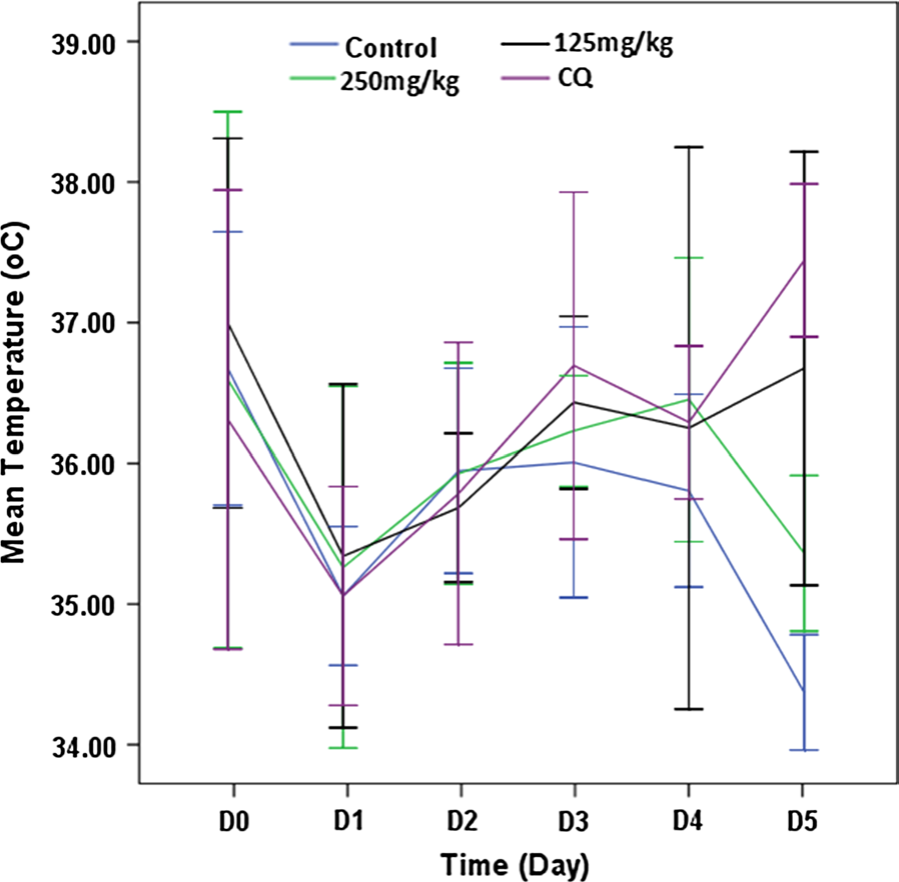
Pattern of rectal temperature of *PbA* infected mice treated with crude extract of seed of *C. molle*

Analysis of rectal temperature over 5 days post-infection revealed that there was a declining pattern after day 4-post-infection. Accordingly, as duration of the infection increased (day 4–5) rectal temperature showed dropping, although significant differences were not observed (Fig. 1).

### Discussion

Traditional medicine is commonly accessible, inexpensive, acceptable and frequently used in large parts of the world including Africa, Asia, and Latin America. According to the estimate of World Health Organization, about 80% of the population in developing countries are still depends on traditional medicine for their primary health care [13]. In most parts of Africa, herbal medicines at home are serving as first line of treatment for malaria related high fever. Recently, due to increasing emergence of drug resistant trait in plasmodium parasite, globally there is urgent need of novel anti-plasmodial medicine [14]. Accordingly, several attempts have been made on evaluation of medicinal plants for their anti-malarial potential. The current study is also part of the attempts on discovery of alternative anti-malarial drug and it’s focused on the evaluation of one of the traditional medicinal plant widely used by local community for treatment of malaria, skin problems and HIV infections (personal observation). Unlike parts of the plant used in other parts of Africa, seed of *C. molle* plant is widely used for all medication purposes in Ethiopia, Gambella region. Practitioners and indigenous people of the region collect seed of this plant, clean it, after grinding in mortar they use the oily juice of the seed extract for treatment of different aliments. Following the traditional practice, methanol extracted juice of seed of the *C. molle* tested for its anti-plasmodial activities.

The preliminary evaluation and screening of the crude extract of *C. molle* seed showed that the plant had good degree of anti-plasmodial activities (63.5%) against murine malaria, *Pb*A. It is reported that *C. molle* has Saponins, Triterpenes, glycosides, phenols, flavonoids, phenolic compounds, saponin, and alkaloids and anthraquinones [15]. Thus, the observed activity could be attributed to its chemical components such as flavonoids, phenolic compounds, saponin, and alkaloids [16]. Some of the studies conducted so far have supported that antiplasmodial activities of plant extracts could be mainly linked to the range of compounds such asterpenes, steroids, coumarins, flavonoids, phenolic acids, lignans, xanthones, anthraquinones, berberine, limonoids, naphthoquinones, sesquiterpenes, quassinoids, indole and quinoline alkaloids [17]. Furthermore, these secondary metabolites are: alkaloids and flavonoids have been known to elicit antiplasmodial activity by blocking protein synthesis in *Plasmodium falciparum* and chelate with nucleic acid base pairing of the parasite respectively [18–20]. Moreover, as saponin, flavonoids and tannins have antioxidant property they can cause oxidative damage to the *Pb*A parasite [21].

According to Rasoanaivo [22], the following inhibition percentages were proposed for in vivo activity of antimalarial extracts at a fixed dose: 100–90% (very good activity); 90–50% (good to moderate); 50–10% (moderate to weak); 0% (inactive). Hence, the observed parasite inhibition of the crude seed extract of *C. molle* fall in the range of good to moderate (90–50%) when it is used at optimum dose. Thus, further fractionation of the crude extracts and re-evaluation of each isolate compound could leads for discovery of novel antimalarial drug with unique mechanism of action.

### Conclusion

The study confirmed that the crude seed extract of *C. molle* plant have anti-plasmodial activity against murine malaria parasite *Pb*A and could improve some clinical symptoms in mice.

## Limitation of the study

As this is a preliminary survey, it has some limitations. The major one is lack of further fractionation of the crude extracts, isolation of the active compounds, and re-evaluation of each isolate for their anti-plasmodial activity.
